# How does COVID-19-related social media usage influence disordered eating? A daily diary study among Chinese adults during lockdown

**DOI:** 10.1186/s40337-023-00952-3

**Published:** 2023-12-19

**Authors:** Bijie Tie, Chengquan Zhu, Jinbo He, Jiang Qiu

**Affiliations:** 1 Center for Studies of Education and Psychology of Ethnic Minorities in Southwest China, Southwest University, Chongqing, China; 2 Key Laboratory of Cognition and Personality (SWU), Ministry of Education, Chongqing, China; 3 Department of Psychology, Sun Yat-Sen University, Guangzhou, Guangdong China; 4 School of Education, Zhengzhou University, Zhengzhou, Henan China; 5 School of Humanities and Social Science, The Chinese University of Hong Kong, Shenzhen, 518172 Guangdong China; 6 Faculty of Psychology, Southwest University (SWU), No. 2 TianSheng Road, Beibei District, Chongqing, 400715 China; 7 Southwest University Branch, Collaborative Innovation Center of Assessment Toward Basic Education Quality, Beijing Normal University, Beijing, China

**Keywords:** Social media, COVID-19-related anxiety, COVID-19-related stress, Psychological distress, Disordered eating

## Abstract

**Background:**

Despite previous studies highlighting the benefits of social media use during the COVID-19 pandemic, particularly under lockdown, limited research has identified the potential detrimental consequences of social media use during lockdown. Therefore, the purpose of this study is to examine the effects of social media on mental health in particular situations and the mechanisms underlying these effects.

**Methods:**

A daily diary protocol was adopted. A total of 96 adults (*M*_*age*_ = 25.90 ± 8.32 years) were recruited from Xi’an, Shaanxi Province, China. COVID-19-related social media use, psychological distress, COVID-19-related stress and anxiety, and disordered eating were measured each day for a week. Multilevel path analyses for the nested data were conducted.

**Results:**

Daily COVID-19-related social media use was positively related to daily disordered eating (*r* = .13 *p* < .001). Furthermore, the multilevel path analysis showed that psychological distress and COVID-19-related stress and anxiety mediated the relationship between COVID-19-related social media use and disordered eating at the within-person level. However, only COVID-19-related-anxiety mediated the relationship at the between-person level.

**Conclusions:**

Our findings contribute to the understanding of social media’s impact during lockdown and provide implications for social media users, social media platform providers, mental health professionals, and governments regarding the correct and sustainable use of social media during the COVID-19 pandemic and in future public health emergencies.

## Abstract (plain language summary)

Our study examined how using social media during the COVID-19 lockdown might be related to individuals’ mental health, particularly stress, anxiety, and disordered eating. Using a daily diary design, we found that individuals with more frequent social media use to get information about COVID-19 exhibited higher stress and anxiety related to the pandemic, higher general psychological distress, as well as higher disordered eating. Multilevel mediation analyses further showed that COVID-19-related anxiety played a mediating role in the connection between COVID-19-related social media use and disordered eating, at the within-person level. These findings underscore the complex impacts of social media use during lockdowns and highlight the importance of managing social media consumption to protect mental health and well-being in such challenging times.

## Introduction

As the coronavirus disease 2019 (COVID-19) pandemic has had a significant inflection rate and negative impact, the World Health Organization designated it as a Public Health Emergency of International Concern in January 2020 [1, 2]. To restrict the spread of the virus, countries implemented rigorous measures such as travel bans and lockdowns [3, 4]. Although lockdown procedures help to prevent the spread of COVID-19, they may increase individuals’ risk of mental health issues [5, 6]. Among the elevated mental health issues, the risk of disordered eating during the COVID-19 lockdowns has been well-documented [7, 8]. Disordered eating include a variety of irregular eating patterns (for example, emotional eating, food restriction, binge eating, and purging) which are highly prevalent in non-clinical populations [9, 10]. However, as shown in a recent review of the impact of the COVID-19 pandemic on disordered eating behaviors, relevant studies were mainly conducted in Western countries [8]. To our knowledge, there is still a paucity of research on how the COVID-19 lockdowns may have impacted Chinese adults’ disordered eating behaviors in China where there are distinct eating and food cultures from Western countries [11]. For example, Chinese people often see eating as a social link because of the societal obligation to establish and maintain connections [11].

In addition, given that social media usage was one of the main means of communication during the COVID-19 lockdown, there have been empirical studies from Western countries revealing the links between social media usage during the COVID-19 lockdown and disordered eating [12–14]. However, previous studies have generally adopted retrospective investigations which may bias the findings due to participants’ memory recall [15]. As suggested [16], diary designs may avoid potential memory bias and is similar to individuals’ real life. Finally, the impact of social media use on mental health, especially in the context of the COVID-19 epidemic, is still being debated, with mixed and inconclusive results [17, 18]. Therefore, the current study used a daily diary method to examine how daily social media usage during a pandemic-induced lockdown may be associated with psychological distress and disordered eating in Chinese adults [19].

### Social media usage during the COVID-19 lockdown

In response to public health emergencies, social media has been used to disseminate timely, locally-relevant health information. Meanwhile, governments and health professionals have aggressively used social media to mitigate the damage caused by health emergencies [20]. For example, social media has been a prominent source of information about COVID-19 [21]. Additionally, in recent years, governments (e.g., in China), in response to the epidemic, have implemented public health measures such as contact tracing, quarantines, and isolation, among others, which may lead to social distancing and consequently an increase in social media usage [22–24].

Prior research has revealed the dark aspects of social media usage during lockdown [25]. In particular, increasing social media use and expanding pandemic information on social media during the pandemic lockdown have contributed to mental health issues. Flaudias et al. [26] have found that exposure to COVID-19-related media coverage was positively correlated with disordered eating and COVID-19-related stress during the lockdown. Furthermore, in recent years, some evidence suggests that social media has become the major source of information about the epidemic during lockdown [21].

In general, social media usage is becoming increasingly frequent during the lockdown and it may have negative effects on mental health.

### COVID-19-related-stress and anxiety and psychological distress during the COVID-19 lockdown

Recent research has indicated that protracted quarantines, limits on public life, and concerns about job security may increase COVID-19-related-stress and anxiety, which may have negative consequences on mental health [27, 28], such as sexual compulsive symptom [29]. The pandemic may also pose a risk to those with disordered eating [30, 31]. Moreover, issues relating to people’s mental health were particularly observed during the period when lockdown measures were imposed. According to the latest report, about 80% of people reported the presence of overeating and most of them experienced psychological distress [12]. As a result, lockdown is likely to increase social media use as a means of communication, potentially increasing the risk of COVID-19-related stress and anxiety, and psychological distress [24, 29, 32, 33].

### The mediating role of COVID-19-related-stress and anxiety and psychological distress

Research has established a link between psychological distress and disordered eating. However, the COVID-19 outbreak presented a unique and unprecedented stressor that may have had distinct influences on disordered eating [31, 33–35]. It’s worth noting that stress and anxiety may lead to different effect on individuals’ emotional problems [36, 37]. Therefore, it is necessary to clarify the specific effects of COVID-19-related stress and anxiety on disordered eating.

According to the stimulus-organism-response (S-O-R) framework, environmental factors interact with personal variables to shape individual behaviors [38]. Therefore, through organismic elements—which include an individual's own emotional and psychological processes when faced with a stimulus—these environmental circumstances might cause particular behavioral intentions and/or actual behaviors [39]. Previous investigations have shown that exposure to COVID-19-related information on social media can increase negative emotions [22, 40]. In addition, social isolation, media exposure, and negative affect have been identified as pathways that contribute to the worsening of disordered eating [41]. In applying this framework to the current pandemic, habitual exposure to negative COVID-19 information on social media can amplify distressing perceptions of the epidemic, potentially leading individuals to develop disordered eating behaviors as a means to distract themselves from psychological distress and COVID-19-related stress and anxiety induced by the lockdown [13, 42, 43]. To explore these complex interactions further, this study aims to bridge this gap by employing a daily diary study design, allowing for the examination of the dynamic relationships between COVID-19-related social media usage, psychological distress, COVID-19-related stress and anxiety, and disordered eating over the course of the lockdown period.

### The present study

To fill the above mentioned gap in the literature, this study uses a week-long diary design on a sample of Chinese individuals who were in lockdown. By studying these variables within the context of the lockdown period, we can gain a better understanding of how social media usage in particular situations affects individuals' mental health and its potential influence on disordered eating among Chinese.

Our working hypothesis is that daily COVID-19-related social media use predicts both direct and indirect daily disordered eating via daily psychological distress, stress, and anxiety. In addition, multilevel modeling allows us to examine whether differences in COVID-19-related social media across persons or between days within people better explain disordered eating. In general, we hypothesize that: (1) COVID-19-related social media usage is positively correlated with disordered eating among Chinese people during a lockdown, (2) COVID-19-related stress and anxiety and psychological distress are positively related to COVID-19-related social media use and disordered eating, and (3) COVID-19-related stress and anxiety and psychological distress are significant mediators of the relationship between COVID-19-related social media use and disordered eating.

## Methods

### Participants and procedure

We recruited participants from community websites (such as WeChat groups) between December 2021 and January 2022 during the Xi’an Province full lockdown by posting the recruiting message for approximately 3 days with details regarding the research protocol (daily surveys for continuous a week and eligibility). As we were only interested in non-clinical samples of adults who were under lockdown, the following eligibility criteria were used: (1) being above the age of 18 years; (2) self-reported having no history of mental disorders (e.g., eating disorders); and (3) being under lockdown. During the recruitment period, 114 people consented to participate in our study and were invited to join our WeChat group. After excluding 11 people who were not under full lockdown (possibly volunteers or community workers), 103 participants remained. All participants read the informed consent form.

The Wenjuanxing online survey platform (www.wjx.cn) was used to collect data. The informed permission form, which was required to access the surveys, was located on the first page of the survey. The initial survey collected a range of socio-demographic information (e.g., age, sex, height, and weight). Every evening at 7 p.m., a WeChat text message was sent to participants with a URL to the survey. By 11 p.m. the same evening, they were directed to submit the survey through the URL. Participants who completed a valid diary entry on at least four out of the seven research days were eligible to receive the full remuneration (¥20). However, four people’s data contained less than 4 days of completed surveys and were excluded. Finally, our study included 645 data points from 96 participants. The sample’s mean age was 25.90 (SD = 8.32) years and BMI based on self-reported height and weight varied from 15.02 to 29.30 kg/m^2^ (M = 21.33, SD = 2.72). Of the participants, 64.6% were women.

All procedures performed in this study involving human participants were in accordance with the ethical standards of Zhengzhou University and with the 1964 Helsinki declaration and its later amendments or comparable ethical standards.

### Measures

In particular, due to the special isolation environment, in order to reduce the fatigue and practice effect of the subjects, we used the short version of each questionnaire as far as possible.

#### Daily social media usage for COVID-19 information

To measure daily COVD-19 social media usage, we adapted the one item for assessing social media usage [44]. Specifically, the item was “Since this time yesterday, how much time did you spend on COVID-19 information websites on social media (i.e., WeChat, WeiBo, DouYin)? Responses had a free-form format and were provided in hours and minutes. For the sake of this analysis, responses were translated to minutes.

#### Daily COVID-19-related stress

In line with previous study, COVID-19-related stress was measured by three items and we adapted these item [45, 46]. These items are scored on a 7-point Likert scale, with higher scores indicating greater degrees of COVID-19-related stress. In this study, the Cronbach’s *α* value for the scale was 0.90.

#### Daily COVID-19-related anxiety

In line with previous studies, COVID-19-related anxiety was used a 1-item to measure [33, 34]. Meanwhile, daily COVID-19-related anxiety, we adapted one item from. Specifically, participants were asked to respond to “How anxious are you about the coronavirus [COVID-19] pandemic today?” on a scale of 1 (not anxious at all) to 7 (extremely anxious).

#### Daily psychological distress

The Short Kessler Psychological Distress Scale (K6) was used to investigate participants’ daily psychological distress [47]. Participants responded to all K6 items, which inquire about the frequency of symptoms experienced over the previous 30 days, using a 5-point Likert scale from 0 (not at all) to 4 (all of the time). Higher scores indicate greater degrees of psychological distress. In line with a previous study, the method of measuring daily psychological distress was adjusted to inquiring about one’s emotions in the preceding 24 h [48]. In this study, the Cronbach’s *α* value for the scale was 0.93.

#### Disordered eating

Same as in previous study, to measure daily changes in eating during lockdown used two items, and adapted these items [13]. Participants were asked: “I have become more preoccupied with food/eating today.” And “I have found it more difficult to regulate or control my eating today.” Participants assessed each item on a scale of 1 (strongly disagree) to 5 (strongly agree). Higher scores indicate greater degrees of change in eating habits. In this study, the Cronbach’s *α* value for the scale was 0.73.

### Data analysis

SPSS 24.0 was used to perform the initial evaluation of the data, including means and standard deviations for each variable. Meanwhile, correlations between variables (including within-level and between level) were estimated.

Next, a two-level nesting structure was used to test all hypotheses. Observations were modeled at Level 1 (within subjects) and participants were modeled at Level 2 (between subjects). Unconditional models including no predictor variables were tested for change in eating to ensure a two-level structure was achieved. We achieved this goal through computing intraclass correlation coefficient (ICC) for each variable through M-plus 8.1. This method captures within- and between-person variability. Small ICC values indicate high within-person variability relative to between-person variability. Variance partitioning results revealed that the intraclass correlation coefficient was 0.45 for the COVID-19-related-social media, 0.59 for COVID-19-related-anxiety, 0.69 for COVID-19-related-stress, 0.75 for psychological distress, and 0.45 for disordered eating, indicating that intraindividual fluctuations explained a significant amount of the variances in the outcome variables. Therefore, the multilevel-modeling approach was appropriate to test our hypotheses.

Finally, we conducted multilevel path analyses in M-plus 8.1 [49] for the nested data (daily responses within individuals), which simultaneously estimated all path coefficients in the full model. Specifically, for MLMs containing observation-level predictors (e.g., COVID-19-related-anxiety, daily psychological distress), models were specified to allow for fixed and random effects. Fixed effects are defined as the average effect of the independent variable on the dependent variable in an individual [50]. Random effects indicate whether there is evidence that a fixed effect varies randomly across different participants within a sample [50].

We tested the model to assess whether there is a positive relationship between individuals’ social media use regarding COVID-19 (including within level and between level) and their daily COVID-19-related-anxiety and stress, psychological distress, and disordered eating. Furthermore, we tested for mediation effects in the event of positive results. Indirect effects were tested using a bootstrap estimation approach with 5000 samples.

## Results

### Preliminary analysis

Table 1 presents the means, standard deviations, and intercorrelations for the study variables. As expected, at the within-person level, individuals’ information browsing about daily COVID-19-related social media usage was positively related to daily COVID-19-related-stress (*r* = 0.21, *p* < 0.001), daily COVID-19-related-anxiety (*r* = 0.19, *p* < 0.001), and daily psychological distress (*r* = 0.26, *p* < 0.001). Daily COVID-19-related-stress (*r* = 0.54, *p* < 0.001) and daily COVID-19-related-anxiety (*r* = 0.63, *p* < 0.001) were positively related to psychological distress. Meanwhile, these three negative outcomes were positively related to individuals’ daily disordered eating (*r* = 0.24–0.63, *p* < 0.001). Moreover, we found the same pattern of correlations between variables at the between-person level.

**Table 1:** Means, standard deviations, and intercorrelations among study variables

Variable	1	2	3	4	5
1. COVID-19-related social media	1	.27**	.32***	.37***	.21*
2. COVID-19-related-anxiety	.19 ***	1	.85***	.64***	.45***
3. COVID-19-related-stress	.21***	.74***	1	72***	.37***
4. PD	.26***	.54***	.63**	1	.30**
5. DE	.13***	.31***	.24***	.24***	1
*M*^a^	55.47	3.45	9.58	11.18	5.76
*M*^b^	54.57	3.45	9.54	11.14	5.76
*SD*^a^	98.03	1.56	4.48	5.22	1.70
*SD*^b^	71.32	1.26	3.84	4.63	1.24

Correlations below the diagonal represent within-person correlations (*n* = 645). Correlations above the diagonal represent between-person correlations (*n* = 96). To calculate between-person correlations, we averaged within-person scores across days

*PD* psychological distress, *DE* disordered eating, *M* mean, *SD* standardized deviation

^a^Within-person

^b^Between-person

*p < .05; **p < .01; ***p < .001

### Multilevel path analysis

**Fig.1:**
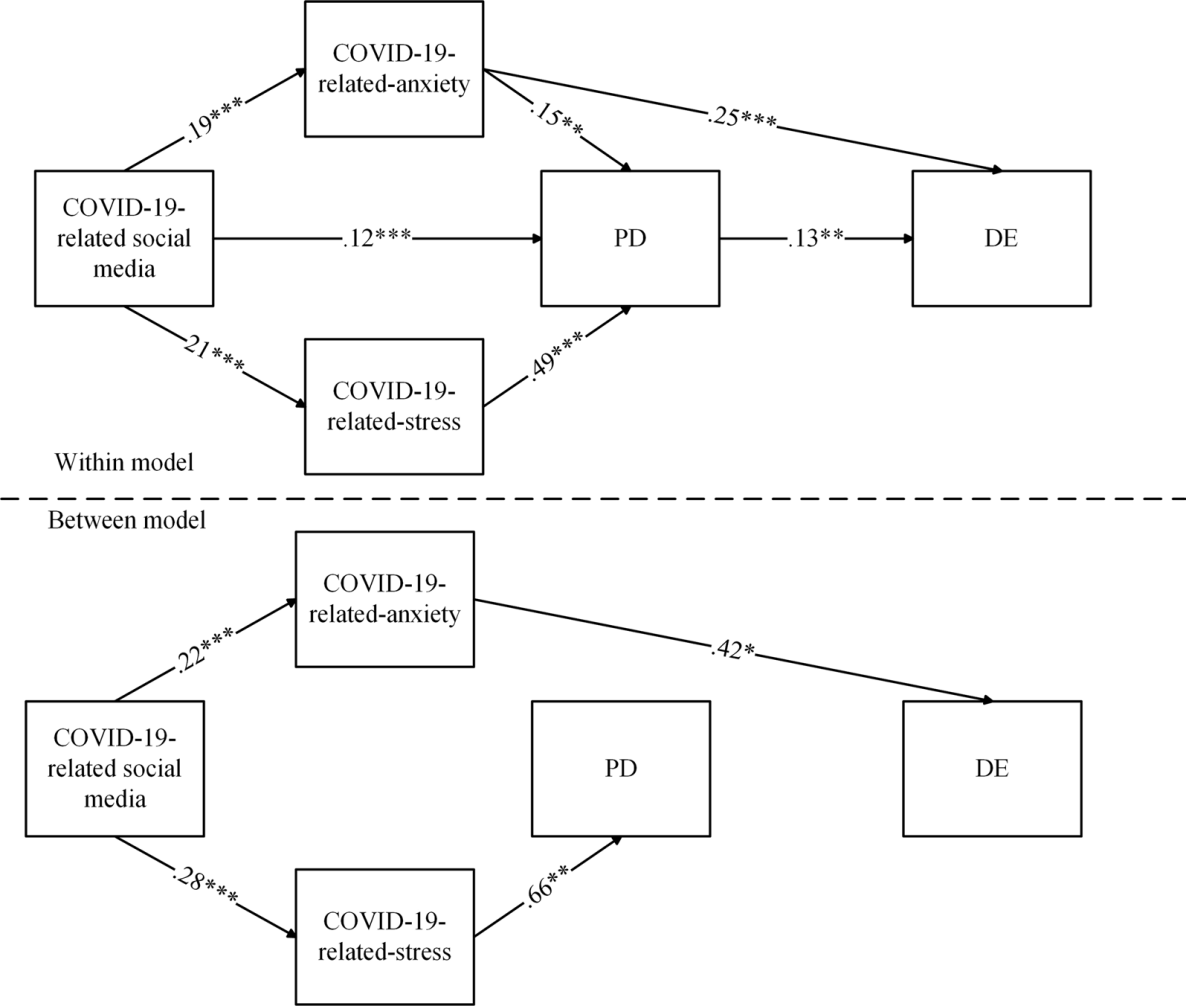
The multilevel path model. *Note*. Control variables were gender, age and BMI. *PD* psychological distress, *DE* disordered eating, *SE* standard error. *p < .05; **p < .01; ***p < .001

Table 2 and Fig. 1 presents the results from the multilevel path analysis that estimated all the path coefficients, including those at the within-person and between-person levels, simultaneously. The results show that individuals’ information browsing about COVID-19 in social media could not directly predict their disordered eating at the within-person level or the between-person level. However, individuals’ information browsing about COVID-19 in social media could affect their disordered eating only through COVID-19-related-anxiety at the between-person level. Meanwhile, at the within-person level, individuals’ information browsing about COVID-19 in social media could affect their disordered eating through COVID-19-related-anxiety and psychological distress, simultaneously. In addition, COVID-19 anxiety could not predict individuals’ psychological distress at the between-person level, but both COVID-19 stress and COVID-19 anxiety could predict psychological distress at the within-person level. Therefore, at the within-person level, the chain mediating roles of COVID-19-related-anxiety/stress and psychological distress were another important link in the relationship between individuals’ information browsing about COVID-19 in social media and their disordered eating.

**Table 2:** Multilevel modeling (MLM) results

Arguments	Antecedent variables: DE	
	Point estimate	*SE*
*Between-person*		
COVID-19-related social media → COVID-19-related-anxiety	.22***	0.08
COVID-19-related social media → COVID-19-related-stress	.28***	0.08
COVID-19-related social media → PD	.12	0.06
COVID-19-related social media → DE	.07	0.07
COVID-19-related-stress → PD	.66**	0.15
COVID-19-related-anxiety → PD	.09	0.16
COVID-19-related-anxiety → DE	.42*	0.17
PD → DE	.07	0.13
COVID-19-related-stress → DE	-.07	0.19
Path: COVID-19-related social media → COVID-19-related-anxiety → DE	.10*	0.10
COVID-19-related-anxiety residual variance	.54***	0.09
COVID-19-related-stress residual variance	.61***	0.09
PD residual variance	.30***	0.05
DE residual variance	.31**	0.06
*Within-person*		
COVID-19-related social media → COVID-19-related-anxiety	.19***	0.04
COVID-19-related social media → COVID-19-related-stress	.21***	0.04
COVID-19-related social media → PD	.12***	0.03
COVID-19-related social media → DE	.07	0.04
COVID-19-related-stress → PD	.49***	0.05
COVID-19-related-anxiety → PD	^.^15**	0.05
COVID-19-related-anxiety → DE	.25***	0.07
PD → DE	.13**	0.05
COVID-19-related-stress → DE	− .03	0.70
Path: COVID-19-related social media → COVID-19-related-anxiety → DE	.05**	0.02
Path: COVID-19-related social media → PD → DE	.02*	0.01
Path: COVID-19-related social media → COVID-19-related-stress → PD → DE	.01*	0.01
Path: COVID-19-related social media → COVID-19-related-anxiety → PD → DE	.004*	0.01
COVID-19-related-anxiety residual variance	.96***	0.05
COVID-19-related-stress residual variance	.91***	0.05
PD residual variance	.58***	0.06
DE residual variance	.87**	0.05

Control variables were gender, age and BMI

*PD* psychological distress, *DE* disordered eating, *SE* standard error

*p < .05; **p < .01; ***p < .001

## Discussion

In this daily diary study, we focused on how COVID-19-related social media usage affects individuals’ disordered eating through COVID-19-related-stress and anxiety, and psychological distress. Overall, the results of this study are consistent with the previous studies that identified lockdown’s negative effect on individuals’ mental health (e.g., disordered eating) [12, 25]. Specifically, more COVID-19-related social media usage during lockdown may arouse higher levels of COVID-19-related-stress and anxiety and psychological distress. Furthermore, these psychological problems may lead to individuals’ disordered eating. More importantly, the present study tested the predictive relationship between these variables through a daily diary study and found that COVID-19-related-anxiety plays a more important role in the relationship between COVID-19-related social media usage and disordered eating than other factors.

Our results suggest that, first, COVID-19-related social media usage during lockdown could not directly predict individuals’ disordered eating, whether at the within-person or between-person levels. Although some studies have shown that the two are closely related [12, 14, 26], our results indicate that the direct condition of individuals’ disordered eating is from other factors. Anxiety, stress, and psychological distress caused by COVID-19-related social media usage has a stronger positive association with disordered eating. We speculated that the mediating role of these three may explain the main association between COVID-19-related social media usage and disordered eating thus leading to an insignificant direct effect in our model.

Second, COVID-19-related social media usage was found to positively predict COVID-19-related-stress and anxiety at both the within-person and between-person levels. This result is consistent with previous studies [26, 29]. This aligns with the S-O-R framework's assertion of environmental factors influencing internal states [39]. However, our results indicate that COVID-19-related-anxiety could predict individuals’ disordered eating but COVID-19-related-stress could not. We speculate that this may be due to the differences between the two and between how individuals respond when faced with the two. Compared with anxiety, stress is more like a precondition of individuals’ emotional problems [36]. Although it has negative attributes, it may not directly cause specific psychological problems, which depends on an individual’s coping style. When stress is caused by external events, people generally manage and deal with it in two ways: positive and negative coping styles. Positive coping styles include problem solving, seeking help, and reconstruction; negative coping styles include escape, denial, and fantasy [51]. People could use positive coping strategies to reduce the impact of stress [52]; however, anxiety is more difficult to manage. Individuals usually relieve anxiety through distracting, exercising, meditating, and sleeping, but these methods are limited under a lockdown environment, which may deteriorate people’s anxiety and cause disordered eating [30, 41]. This variation in response within the organism component of the S-O-R framework is critical in understanding individual differences in coping with pandemic-related stressors.

Third, at the between-person level, only COVID-19-related-anxiety could mediate the relationship between COVID-19-related social media usage and disordered eating, which implies that the anxiety caused by social media usage in lockdown situations can be integral to people’s change in eating. Given that our mediation models included multiple covariates, including COVID-19-related stress and psychological distress, the nonsignificant effects suggest that COVID-19-related anxiety was more significant between COVID-19-related social media usage and disordered eating than COVID-19-related stress and psychological distress. This result is an important guide for the government’s segregation management policy. This highlights the significant role of specific organismic responses in the framework, underlining the importance of considering individual psychological factors in the stimulus–response relationship. The government should verify COVID-19 information available on social media to ensure that the public is informed of news that is scientific, true, and accurate. Regarding the available COVID-19 information, it is important for the government to consider which kinds of information brings the most anxiety to people. If it is rumors about COVID-19 that cause the most anxiety, then strict management of the online environment can significantly reduce individuals’ anxiety and thus protect their mental health. Furthermore, more targeted advice on mental health protection for people in isolation can then be provided.

In this post-pandemic period, as governments ease or lift quarantine measures, the lasting influence on individual lifestyles, especially in social media usage, is evident [53]. For instance, there has been an increase in using social media for health-related information [54], highlighting its evolving role in the dissemination of medical services post-pandemic. However, the increased reliance on social media simultaneously highlights the risk of its excessive use [55, 56]. This dual aspect of social media usage, serving both as a tool for health information and as a factor that could negatively impact mental and physical health, calls for a balanced approach. Future research should focus on the long-term effects of these behavioral changes, exploring strategies for fostering healthier online habits.

This study has some limitations. The measurements in this study are all from the self-report of the participants; other objective indicators should be included in future studies. In addition, we relied on only two questions to measure disordered eating because our study was done during a lockdown. This narrow scope may not fully reflect the wide range of disordered eating behaviors that people may exhibit during lockdown scenarios. Future research should examine using a broader set of measures to comprehensively measure multiple disordered eating behaviors in people under lockdown. Furthermore, the participants in this research were non-clinical adults, and it is unknown if the suggested model can be used for clinical samples from the Chinese community (e.g., those with eating disorders) or children and adolescents. Finally, even though the current research employed a daily diary, its time frame could not have been long enough. Despite the fact that individuals are only alone for short periods of time, the effects of isolation on a person’s mental health can last for much longer. Through longitudinal study, this topic can be further investigated.

## Conclusion

This study provides empirical evidence that COVID-19-related social media usage during lockdown has a negative effect on individuals’ eating. Furthermore, our results indicate that only COVID-19-related anxiety could mediate the relationship between COVID-19-related social media usage and disordered eating. This finding calls for more attention to be paid to the implications of anxiety on individuals’ mental health when they are in a lockdown.

## Data Availability

The data used in the current study is available from the corresponding author upon reasonable request.
